# Novel Virulence Factors Deciphering *Klebsiella pneumoniae* KpC4 Infect Maize as a Crossing-Kingdom Pathogen: An Emerging Environmental Threat

**DOI:** 10.3390/ijms232416005

**Published:** 2022-12-15

**Authors:** Min Huang, Pengfei He, Pengbo He, Yixin Wu, Shahzad Munir, Yueqiu He

**Affiliations:** 1 State Key Laboratory for Conservation and Utilization of Bio-Resources in Yunnan, Yunnan Agricultural University, Kunming 650201, China; 2 College of Agronomy and Life Sciences and Engineering Research Center for Urban Modern Agriculture of Higher Education in Yunnan Province, Kunming University, Kunming 650214, China

**Keywords:** genome, crossing-kingdom pathogen, *Klebsiella pneumoniae* KpC4, virulence prediction, novel genes

## Abstract

*Klebsiella pneumoniae* is not only a human and animal opportunistic pathogen, but a food-borne pathogen. Cross-kingdom infection has been focused on since *K. pneumoniae* was identified as the pathogen of maize, banana, and pomegranate. Although the pathogenicity of *K. pneumoniae* strains (from ditch water, maize, and human) on plant and mice has been confirmed, there are no reports to explain the molecular mechanisms of the pathogen. This study uncovered the *K. pneumoniae* KpC4 isolated from maize top rot for the determination of various virulence genes and resistance genes. At least thirteen plant disease-causing genes are found to be involved in the disruption of plant defense. Among them, *rcsB* is responsible for causing disease in both plants and animals. The novel sequence types provide solid evidence that the pathogen invades plant and has robust ecological adaptability. It is imperative to perform further studies on the verification of these KpC4 genes’ functions to understand the molecular mechanisms involved in plant–pathogen interactions.

## 1. Introduction

*Klebsiella pneumoniae* is a notorious human and animal opportunistic pathogen for its serious nosocomial, community or healthcare-associated infections, and intractable multiple antibiotic resistance in the medical field [1,2]. It causes pneumonia, urinary tract infection, biliary tract infection, meningitis, bacteremia, and bloodstream infection in immunocompromised people and malnourished children [1,3], even in immunocompetent adults [4]. More and more attention has been drawn to the convergence of hypervirulence with multidrug-resistance of clinical *K. pneumoniae* [5].

Whereas, except on mucosal surfaces of mammals, *K. pneumoniae* also ubiquitously resides in the environment (soil, water, etc.) [6] and the surface of raw vegetables. Currently, studies revealed that *K. pneumoniae* frequently has been detected in fresh lettuce, arugula, cucumber, tomato, spinach, carrot, parsley, coriander, jute, and some herbs, even processed juice and canned food [7,8]. Symptoms of food poisoning such as abdominal pain, diarrhea, and vomiting appear after eating these foods contaminated with *K. pneumoniae* [8]. *K. pneumoniae* can reach and adhere to the surface of the plant, then colonize and undergo internalization. Shilpi et al. [7] revealed that *K. pneumoniae* (10^8^ cfu/mL) can colonize on tomato leaves with no morphological or pathological changes. It is also likely to be related to the capsule and lipopolysaccharide of *K. pneumoniae* and its biofilm formation. As a food-borne pathogen, *K. pneumoniae* takes the plant as a reservoir or vector back to its human and animal hosts. The epiphytic or endogenous growth of *K. pneumoniae* is an important part of its life cycle. However, as a cross-kingdom pathogen which can infect plants, *K. pneumoniae* KpC4 can actively invade and exhibit cross-kingdom pathogenicity in maize [9], banana [10], and pomegranate [11], and cause top rot, leaf spot, and blight symptoms, respectively, in the field naturally, and cause sorghum leaf spot by artificial inoculation [12]. Huang et al. have illustrated that KpC4 and other *K. pneumoniae* strains still can survive in different environments and variant harsh conditions, such as soil, different quality water, dry filter paper, maize phylloplane, interior, kraurotic maize plant debris, poor nutrients, exposure to ultraviolet radiations, and unfit temperatures [12]. From the perspective of ecology, the extensive ecological adaptability of *K. pneumoniae* is the cross-kingdom infection mechanism of KpC4.

There are several virulence factors such as capsule, lipopolysaccharide, fimbriae, siderophores, outer membrane proteins, and type VI secretion systems [3,13] associated with human and animal infections that have been well described. Whether these virulence factors are also the agents of plant diseases requires further study. Previously, studies revealed that, under the experimental conditions, some human opportunistic pathogenic bacteria such as *Salmonella enterica*, *Pseudomonas aeruginosa*, and *Enterococcus faecalis* can infect plants using same virulence factors with humans and animals [14]. Interestingly, *K. pneumoniae* KpC4 has been demonstrated that it also has the same pathogenicity as the clinical strain Kp138 (K1 serotype) to infect mice; and the clinical strain (Kp138) and environmental strain E4 (K1 serotype) can cause maize top rot [9,12]. It is likely that KpC4 uses the same or different strategies (disease-related factors) to infect plants. Bioinformatics plays an important role in evaluating potential virulence factors of the microbes. In silico approaches, such as complete genome sequencing, identification of crucial genes of the pathogen responsible for the virulence and searching the homologous proteins between *K. pneumoniae* and plant pathogenic bacteria, could be used to reveal the pathogenesis of crossing-kingdom infection of KpC4. However, these hypothetical pathogenic mechanisms require further phenotypic and functional verification. To gain insights into the genetic elements involved in maize infection and cross-kingdom infection mechanisms, complete genome sequencing of *K. pneumoniae* KpC4 was performed to explore the hidden molecular mechanisms involved in its interactions inside the host.

## 2. Results

### 2.1. Genome Features

**Figure 1:**
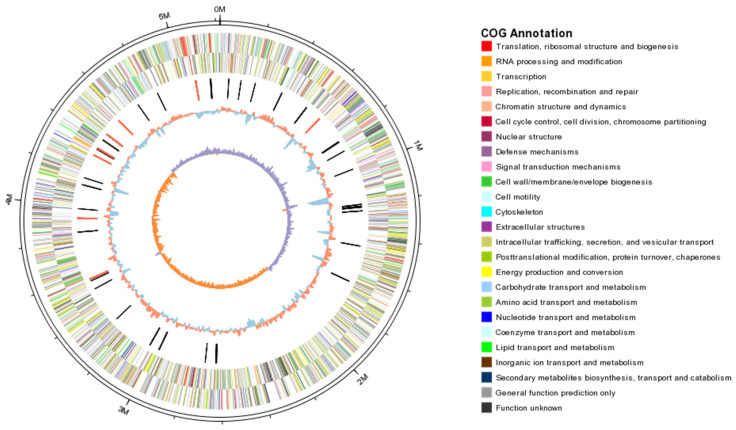
Circular map of the chromosome of *K. pneumoniae* KpC4. From the outside in, the first and second circles show the predicted protein-encoding regions on the plus and minus strands, by role, using the colors for the COG functional categories (http://www.ncbi.nlm.nih.gov/COG/grace/fiew.cgi (accessed on 3 July 2020)). The third circle shows tRNA (black) and rRNA (red). The fourth circle shows the GC content (red indicates > mean value, blue indicates < mean value). The fifth circle shows the GC skew (GC skew = (G − C)/(G + C); purple indicates > 0, orange indicates < 0).

The genome of KpC4 is composed of a circular chromosome of 5,218,784 bp (Figure 1) with an overall G+C content of 57.26%, which is similar to previous reports of *K. pneumoniae* NTUH-K2044 (57.7%), MGH 78578 (57.5%), and 342 (57.3%) [15]. The chromosome encodes 4912 putative coding sequences (CDS) representing 87.3% coding density, and the total length of the coding region is 4,556,229 bp with G+C content of 58.87%. The average chromosomal gene length was found to be 927 nucleotides. A total of eighty-three tandem repeats and five transposons were found. Consistent with MGH 78578, Kp13, and NTUH-K2044, 86 tRNA genes with specificities were identified. The number of rRNA genes (16s-23s-5s) were eight, eight, and nine, respectively, which is same as NTUH-K2044 [16]. The characteristics of the genome and its comparison with other genomes of related *K. pneumoniae* are shown in Table 1.

**Figure 2:**
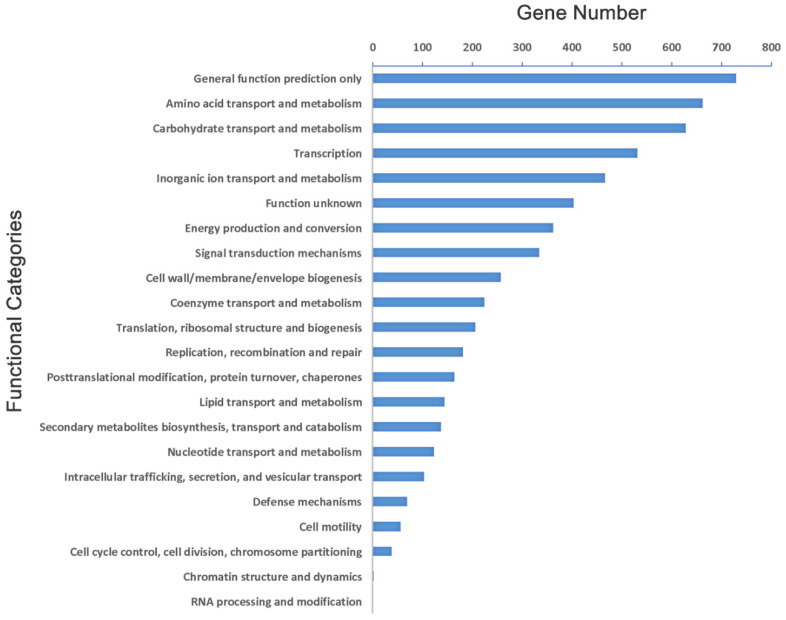
Analysis of COG functional of genome features of KpC4.

The preliminary analysis of the genome suggests that 4509 (91.8%) of the CDSs can be assigned biological role categories, while 403 (8.2%) have been annotated as enzymes of unknown function. Analysis of cluster of orthologous group (COG) showed that there were 729 genes with general function, 662 genes involved in amino acids transport and metabolism, while only a single gene involved in RNA processing and modification (Figure 2).

### 2.2. The Survival Mechanism of Strain KpC4 in Plants

*K. pneumoniae* is ubiquitous and has a wide host range in nature. There are alternative plant hosts besides humans and animals [12]. *K. pneumoniae* developed a survival strategy to overcome the stage of nutrient deficiency and adversity stress in the long process of evolution and adaptation. For example, *K. pneumoniae* could encode various transporters involved in the absorption of carbohydrates, amino acids, and iron, and enzyme systems which decompose some undegradable macromolecules in plants, or improve the tolerance to some toxic substances, such as heavy metals and chemical pesticides, in the environment.

### 2.3. Carbohydrate Metabolism

Like most microbes, KpC4 could use carbohydrates to produce the substances necessary for its own growth and reproduction. Carbohydrate is a kind of important organic matter widely existing in plants, accounting for more than 50% of plant dry weight. The main components of the plant cell wall are cellulose, hemicellulose, pectin, and lignin.

Cellulose is the main component of the plant cell wall and the most abundant carbohydrate in the biosphere, which has a chemical structure composed of a straight chain macromolecular compound of glucose joined by a β-1,4 glucosidic bond. The decomposition of cellulose in organisms is mainly by enzymolysis. Three enzymes, endoglucanase, exo-β-1,4-glucanases, and beta-glucoside (cellobiohydrolases), combine to break down cellulose. There are at least two genes (endoglucanase/endo-1,4-D-glucanase, KpC4_4548 and endoglucanase Y, KpC4_4556) in the KpC4 genome, which were confirmed to have the ability to degrade highly ordered forms of insoluble cellulose. Additional genes encoding enzymes with specificity towards 1,4-β-glucosidic bonds which most likely act by hydrolyzing short cellooligosaccharides include: interperimental *bglX* (beta-D-glucoside glucohydrolase, KpC4_0991), and similar *bglA* (6-phospho-beta-glucosidase, KpC4_0194), *bglB* (KpC4_0496, KpC4_0706, KpC4_0983), *bglG* (Beta-glucoside bgl operon antiterminator, BglG family, KpC4_0981; transcription antiterminator BglG, KpC4_3805), *bglH* (aryl-phospho-beta-D-glucosidase, KpC4_0983, KpC4_0496; cryptic outer membrane porin BglH, KpC4_4311), *bglC* (Aryl-phospho-beta-D-glucosidase, KpC4_1604). Additional β-glucosidase (KpC4_0991, KpC4_0331, KpC4_0480), 6-phospho-beta-glucosidase (KpC4_0706, KpC4_0983, KpC4_1604, KpC4_1875, KpC4_2197, KpC4_2324, KpC4_0194, KpC4_2726, KpC4_3798, KpC4_0301, KpC4_4418, KpC4_0480, KpC4_0496), aryl-phospho-beta-D-glucosidase (KpC4_1604). Although the gene encoding the cellobiohydrolases is absent, the enzymes above could decompose cellulose to produce oligosaccharides and cellobiose, and eventually be hydrolyzed to glucose for cell life.

Genomic analysis revealed that KpC4 has ability to catalyze many different forms of hemicellulosic substrates into fermentable sugars. For instance, the KpC4 genome possesses the genes to metabolize xylan, arabinose, and xylose, including duplications of *xylA* (Xylose isomerase, KpC4_4508, KpC4_3736) and *xylB* (xylulokinase, KpC4_4509, KpC4_1019) which are responsible for creating the phosphorylated derivative, D-xylulose 5-phosphate. β-1,4 mannanase (KpC4_0479) hydrolyzes mannosan by acting on the mannose bond of the mannitosan main chain. Xylanase (KpC4_0981, KpC4_4312) can cut the β-1,4 glucosidic bonds from the main chain of xylose to form oligoxylose and a small amount of xylose [18], which plays an important role in the hydrolysis of xylanase. Both enzymes are endonucleases, which can randomly cut off the glucosidic bond of the main chain to generate oligosaccharides. Then, the oligosaccharide is digested by different glycosidases, including β-mannosidase (KpC4_2183) or β-xylosidase (KpC4_1060, KpC4_0106, KpC4_3738) with external cutting. While glycoside hydrolases, such as galactosidase (KpC4_3154), α-galactosidase (KpC4_3944, KpC4_4237), β-galactosidase (KpC4_1604, KpC4_3153, and KpC4_3837), and β-D-galactosidase (KpC4_1958), can hydrolyze galactoside into monosaccharides. In addition, the genome also possesses β-1,4-xylosidase (KpC4_3738) responsible for the hydrolysis of 1,4-β-D-xylans, α-N-arabinofuranosidase (KpC4_3739), and arabinogalactan endo-1,4-beta-galactosidase (KpC4_3154). Arabinofuranosidases work synergistically with xylanases to degrade xylan to its component sugars.

Pectin is also a major component of plant cell walls. Pectinase is often secreted by pathogenic bacteria to degrade the cell wall during infection. Essential in pectin metabolism of oligogalacturonate lyase (pectate lyase family 22 (EC:4.2.2.6), KpC4_0283) and its oligogalacturonate transport system permease protein (OgtB, KpC4_0294), pectin methylesterase (KpC4_2787) can be found in the KpC4 genome. Pectin methylesterase can utilize the intermediate layer and the pectin of the cell wall, and eventually lead to the death of host tissue, playing a vital role in the pathogenicity of the *Pectobacterium atrosepticum*. The pectinesterase of *Erwinia carotovora* and *Aspergillus niger* is related to the segregation and soft rot of plant tissue. Furthermore, the KpC4 genome also encodes genes capable of degrading the α-linked glucans (primarily 1,4-α and 1,6α-linkages) of plant starches as well as the degradation of low-molecular-weight carbohydrates produced from their breakdown such as maltodextrins, pullulan, and D-galacturonate (Additional Table S1).

### 2.4. Aromatic Compounds Degradation

Aromatic compounds, common in plant cells, are widely and plentifully distributed in the environment and are mainly derived from the decomposition of plant lignin in nature [19]. These compounds serve as signals for plants to perceive when bacteria approach, which may play an important role in colonizing plants [20]. Genomic analysis confirmed KpC4 has the potential to oxidatively catabolize various low-molecular-weight aromatic compounds, most of which are derived from lignin degradation, including ferrulic acid, vanillate (KpC4_1900, KpC4_1901, KpC4_1649, KpC4_1529), 2-chlorobenzoate (KpC4_1702- KpC4_1700), the central aromatic ring metabolites, protochatechuate, and catechol [21,22]. Ring cleavage is mediated by 3,4-protocatechuate dioxygenase (KpC4_1617-KpC4_1618) and catechol 1,2-dioxygenase (KpC4_1699) in the protocatechuate pathway and catechol ortho cleavage pathway, respectively. A complete β-ketoadipate pathway (KpC4_2033-KpC4_2031) of the KpC4 genome is responsible for further decomposition of the ring cleavage degradation products to TCA cycle intermediates [21,22]. Additionally, genomic analyses revealed that the KpC4 genome contain genes that degrade certain aromatic compounds by reduction or non-oxidative decarboxylation, such as several 4-hydroxybenzoate decarboxylase enzymes (KpC4_0453-KpC4_0452), 3,4-dihydroxybenzoate decarboxylase (KpC4_2404), and 3-octaprenyl-4-hydroxybenzoate decarboxylase (KpC4_4106), mainly for decarboxylation of 4-hydroxybenzoic acid, generation of phenol, and CO_2_.

### 2.5. Survival against Plant Defenses

#### 2.5.1. Evade the Plant’s Defense System

Pathogenic bacteria, in the process of infecting host plants, establish parasitism with plants, damage the normal physiological and metabolic function of host cells, regulate the adhesion, infection, colonization and expansion of plants, and finally cause the plants to show symptoms. On the other hand, plants also utilize various non-specific strategies to defend threats from bacteria, viruses, and fungi, including the production of reactive oxygen species (ROS), such as superoxide, hydrogen peroxide, hydroxyl radical, hydroperoxyl radical, nitric oxide (NO), and phytoalexins [23,24]. The genome of KpC4 encodes a powerful enzyme system that protects itself from all three plant defense mechanisms. There are three superoxide dismutases, *soda* (KpC4_4223), *sodB* (KpC4_1571) and *sodC* (KpC4_1580), as well as three catalases (KpC4_1752, KpC4_2322, KpC4_2412), one thioredoxin reductase (KpC4_2652), two glutathione peroxidases (KpC4_1392, KpC4_2374), sixteen NADH dehydrogenases (KpC4_0881-KpC4_0892, KpC4_2461, KpC4_0332, KpC4_4883, KpC4_0489), a NADH dehydrogenase transcriptional regulator (KpC4_1768, LysR family NADH dehydrogenase transcriptional regulator, KpC4_0879), four peroxiredoxin OsmC (KpC4_1741, KpC4_0715, KpC4_2915, KpC4_3234), two thiol peroxidases (KpC4_0715, KpC4_2194), two predicted iron-dependent peroxidases (KpC4_0762, KpC4_2514), one hydroperoxide reductase (encoded by ahpC, KpC4_2915, KpC4_3234, and ahpF, KpC4_2914), and thirteen glutathione S-transferases (GST) (KpC4_0867, KpC4_0868, KpC4_1592, KpC4_1674, KpC4_0110, KpC4_2703, KpC4_2975, KpC4_3211, KpC4_3337, KpC4_3987, KpC4_4497, KpC4_4571, KpC4_4803) (compared to seven in *E. coli* K12, twelve in Kv342), which protects itself against ROS. In addition, the genome encodes flavodoxin reductase (ferredoxin-NADPH reductase family 1, KpC4_2103, KpC4_2598) (compared to one in Kv342 [17]) and anaerobic nitrate reduction operon (KpC4_0505, KpC4_0503-KpC4_0502), which make KpC4 capable of detoxification of the free radical nitric oxide [25]. Additionally, it also possesses the RND-family AcrAB and efflux pump (multidrug transport protein, outer membrane (RND family), KpC4_1034, KpC4_1415, KpC4_2792, KpC4_4391, KpC4_3139, KpC4_3007, KpC4_3721, KpC4_0574), multidrug transporter AcrB (KpC4_2793, KpC4_3140), MDR efflux pump AcrAB, transcriptional activator MarA (KpC4_1941), and MDR efflux pump AcrAB transcriptional activator RobA (KpC4_3605) necessary for the export of apple tree pytoalexins by *E. amylovora* [26].

#### 2.5.2. Plant-Induced and Associated Genes

Many studies have shown that plant-inducible genes of bacteria can be induced and expressed in vivo when bacterial cells colonize or grow on plants. These genes not only exist in plant pathogens [27], but also in animal pathogens [17]. Several plant-induced genes are involved in the bacterial response to oxidative stress and DNA damage caused by the plant defense response, as well as DNA damage repair. Genomic analysis showed that some genes in the genome of KpC4 are homologous with these plant-induced genes. For example, some amino acids and nucleotide biosynthesis genes of KpC4 were highly homologous with the induced genes of *Ralstonia solanacearum* and *Pseudomonas syringae* pv. *tomato* when they colonized the host plants. Genes with this trait include CTP synthase (*pyrG*, KpC4_0414), acetyl-CoA acetyltransferase (KpC4_0281, KpC4_1958), amidophosphoribosyl transferase (*purF*, KpC4_0855), argininosuccinate synthase (*argG*, KpC4_4851), diaminopimelate decarboxylase (*lysA*, KpC4_0298), and acetolactate synthase large subunit (*ivlI*, KpC4_3516) [28,29].

Some assumed stress response genes expressed in *R. solanacearum* which in response to plant defense during colonization in host plants were also found in KpC4. For example, a regulatory protein of the adaptive response (*ada*, KpC4_0926), excinuclease ABCD (*uvrA*, KpC4_4009, *UvrB*, KpC4_2773, *uvrC*, KpC4_1150, *uvrD*, KpC4_4137), DNA-damage-inducible protein F (*dinF*, KpC4_4023), fumarate hydratase (*fumC*, KpC4_2057), and acriflavin resitance protein A (*acrA*, KpC4_3139) [29]. Among these, *ada* is essential for transcriptional activation of genes that are involved in the adaptive response to DNA methylation damage and activated by NO [30]. *uvrABCD* are involved in UV-induced DNA repair from damage recognition to repair in concert. In addition, *uvrA* is also involved in the repair process of DNA damage induced by H2O2 and toxic chemicals indicating that this gene can protect bacteria from DNA-damaging compounds produced by plants [31]. These oxidative response genes are not limited to DNA repair pathways. While *fumC*, apart from being part of the TCA cycle, has been found to be efficiently expressed when superoxide radicals accumulate [32]. Another form of fumarate hydratase, encoded by *fumA*, is inactivated under oxidation [32,33]. The early stage of the plant defense response mainly involves ROS accumulation, so the induction of oxidation-stress-related genes indicates that bacteria, such as KpC4, actively evade defense mechanisms when colonizing plants. HRP-dependent type III effect of protein was also identified in the genome of KpC4, but no non-toxic proteins.

#### 2.5.3. Plant Disease-Causing Genes

**Figure 3:**
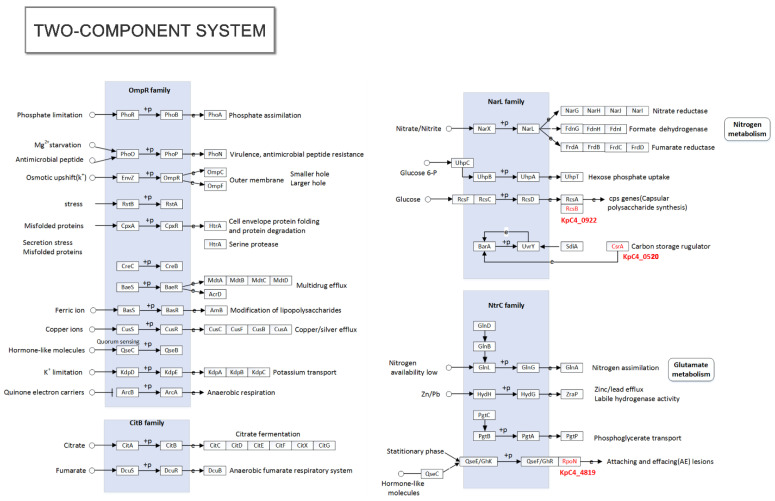
Two-component system of KpC4.

The two-component system is the most widely existing in bacteria as a combined mechanism of stimulating response to environmental change and response. The two-component system of KpC4 is composed of OmpR, CitB, NarL, and NtrC (Figure 3), including sensing and regulating nutrients (NarX-NarL), anaerobic respiration (ArcB-ArcA), osmotic pressure (EnvZ-OmpR), antibiotic (BaeS-BaeR), and gene clusters important for controlling cell growth, virulence (PhoO-PhoP), biofilms (CpxA-CpxR), and quorum sensing (QecC-QseB).

Notably, thirteen protein sequences of plant pathogenic bacteria that are more than 70% the same as the KpC4 in the PHI (pathogen host interactions) database were found (Table 2). The thirteen genes are mainly the pathogenic genes of bacteria of *Pectobacterium*, *Erwinia*, *Xanthomonas,* and *Pantoea*. Among them, the *rcsB*, *rpoN* of *E. amylovora,* and *rsmAXoo* of *Xanthomonas oryzae* pv. *oryzae* were 92.09%, 84.07%, and 82.14% identical with the query of KpC4 (KpC4_0922, KpC4_4819, and KpC4_0520) respectively, which were all related to the two-component system (Figure 3).

**Figure 4:**
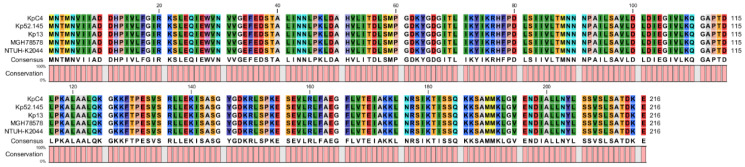
RcsB multi-sequence comparison results of KpC4 and *K. pneumoniae* clinical strains.

Interestingly, the *rcsB* gene, the pathogenic factor of *E. amylovora* (pear fire blight), is a transcriptional regulator of capsular polysaccharide synthesis [34]. Auxiliary transcriptional regulators (*rcsA*), are highly conserved in many bacteria, including human and animal pathogenic bacteria (*E. coli, K. pneumoniae*), as well as some plant pathogens, such as *E. amylovora* and *Pectobacterium atrosepticum* (rotting of tubers of potato). The alignment results showed that the homology of the RcsB protein sequences between KpC4 and *K. pneumoniae* clinical strains MGH78578, NTUH-K2044, Kp13, and Kp52.145 were 100% (Figure 4), and 100 *K. pneumoniae* strains on NCBI were also 100%.

It has been proved that RcsB has a positive regulatory effect on the high mucosity and biofilm formation of *K. pneumoniae* NTUH-K2044 [35]. These two characteristics are not only the important pathogenic factors of animals, but also of plants. The *rcsB* gene of *E. amylovora* was considered as the pathogenic gene of pear fire blight because it facilitates the synthesis of capsular polysaccharide. The capsule creates a favorable microenvironment for the bacterial cells and prevents it from water stress and plant defense reactions, as well as making the plants susceptible to present typical symptoms, such as wilting, necrosis, even bacterial ooze (the main component is capsule polysaccharide).

### 2.6. Pathogenicity of KpC4

Genomic analysis showed that KpC4 encodes the main virulence factors possessed by *K. pneumoniae*-infected humans and animals.

#### 2.6.1. Capsule

**Figure 5:**
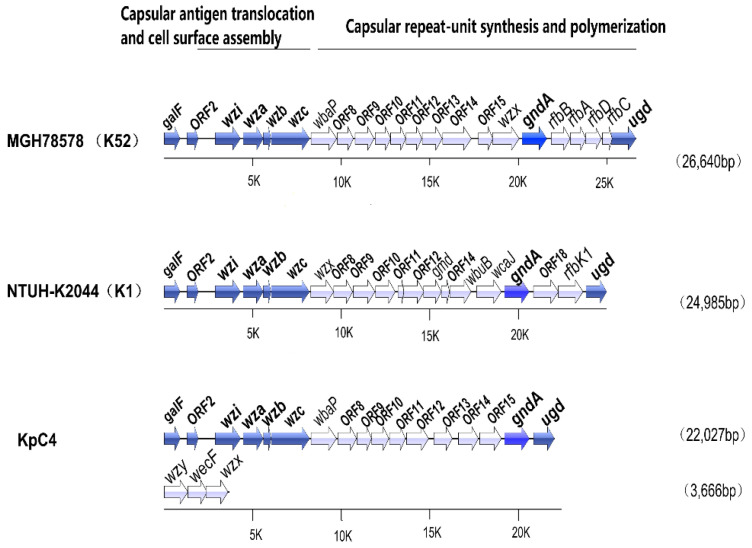
Comparison of capsular polysaccharide synthesis (cps) gene clusters between *K. pneumoniae* KpC4, NTUH-K2044 (K1), and MGH78578 (K52). The locations and orientations of the open reading frames (ORFs) are indicated by arrows. ORFs are cited in order, ORFs with homologs are cited by putative gene names, and those without homologs are cited as ORFs and are numbered. Starting from *galF*, considered to be no. 1 (ORF** in KpC4, ORF in K2044, and ORF* in MGH78578). Solid arrows refer to ORFs with significant similarity (the aligned genes with scores > 200 and expect values < e^−50^ in the BLASTP search), and unshaded arrows indicate the ORFs with low similarity between any two strains (the homolog could not be found even when the expected threshold was set to be 10 in the BLASTP search). Nos. beneath each axis denote positions in kilobases.

The *cps* (capsular polysaccharide synthesis) gene cluster of KpC4 consists of 17 genes (Figure 5, Additional Table S1), including several genes related to capsule synthesis. Similar to NTUH-K2044 and MGH78578, *cps* gene clusters of KpC4 are located downstream of *galF* and *ORF2*, and *wzi (orfX)-wza*-*wzb*-*wzc* genes cluster together. *galF*, *OFR2*, *wzi*(*orfX*), *wza*, *wzb*, *wzc,* and *gnd* are conserved in *K. pneumoniae* strains of different serotypes. *GalF* (UTP–glucose-1-phosphate uridylyltransferase, KpC4_1048), *ORF2* (acid phosphatase, KpC4_1049), and *gnd* (gluconate-6-phosphate dehydrogenase, KpC4_1063) are involved in carbohydrate metabolism [36]. *wzi* (capsule assembly protein, KpC4_1050) encodes a surface protein that is related to the capsule adhesion to the outer membrane; bacteria could not form capsule if Wzi is deficient. *Wza* (polysaccharide export protein, KpC4_1051) is related to surface assembly. *Wzb* (KpC4_1052) encodes low molecular weight protein-tyrosine-phosphatase. *Wzc* (KpC4_1053) encodes a tyrosine protein kinase, which is responsible for translocation and surface assembly of the capsule [37,38].

The *cps* loci of K2044(K1), KpC4, MGH78578(K52) encode different serotypes, and some functions are similar, such as encoding glycosyl transferase (Additional Table S1). However, there are distinct differences in the sequences of orthologous genes and the deficiency or acquisition of genes. The *wbap-ORF15* domain of KpC4, *wzx-rfbp* of K2044, and *ORF8*–*wzx* of MGH78578(K52) show lower similarity (Figure 5), probably as these non-conservative regions are unique to the different serotypes. For example, *wbaP* (KpC4_1054) and *wbaP* of MGH78578 are specific in some serotypes, while *rfbP* is relatively specific in certain serotypes. *wzy* in the genome of KpC4 is highly homologous with the gene of MGM78578, which is related to the polymerization of capsular polysaccharide. *wzx* (O-antigen translocase, KpC4_4154) and *wzy* (common antigen polymerase, KpC4_4152) cluster individually in the genome of KpC4. Additionally, *magA* (one kind polymerase similar to *wzy*) involved in capsular formation, could not be found in the genome of KpC4, which coincides with the experimental result of KpC4 of the non-K1 serotype.

The synthesis of KpC4 capsular polysaccharide was mainly encoded and controlled by *rcsF* (KpC4_3382), *rcsC* (KpC4_0921), *rcsD* (KpC4_0923), *rcsA* (KpC4_1140), and *rcsB* (KpC4_0922), which belong to the NarL family of transcriptional regulation [39].

#### 2.6.2. Lipopolysaccharide

Lipopolysaccharide (endotoxin), the component of the cell wall of Gram-negative bacteria, is composed of lipid A, core oligosaccharide, and O antigen polysaccharide, encoded by *lpx, waa,* and *wb* gene clusters, respectively [40,41]. The lipid A biosynthetic pathways of KpC4 are controlled by enzymes which encoded by *lpxA* (UDP-N-acetylglucosamine acyltransferase, KpC4_3397), *lpxC* (UDP-3-O-acyl N-acetylglucosamine deacetylase, KpC4_3497), *lpxB* (lipid-A-disaccharide synthase, KpC4_3396), *lpxD* (UDP-3-O-(3-hydroxymyristoyl)-glucosamine n-acyltransferase, KpC4_4042) and *lpxK* (tetraacyldisaccharide 4′-kinase, KpC4_2620).

The whole *Waa* gene cluster is located between *kbl* and *coaD*, of which *E. coli* k-12 contains 12 genes [42]; O-antigen, located in the outermost layer of lipopolysaccharide, is composed of oligosaccharide repeating units. *Wb* gene clusters (*manC*, *manB*, *wzm*, *wzt*, *wbbD*, *wbdA*, *wbdB*, *wbdC*, *hisI*) usually include biosynthetic genes, such as active sugar, glycosyltransferase, O antigen polymerase, and O antigen output protein [43]. The whole *Waa* gene cluster of KpC4, including 13 genes (Table 3), is located between *kbl* and *coaD*, while *E. coli* k-12 contains 12 genes [42]. Interestingly, the *wb* gene clusters encoding o-antigen polysaccharide were not found in the genome of KpC4. Perhaps the variation of *wb* gene clusters could be related to the biosynthesis of the O-antigen due to the high chemical variability of the o-antigen [43].

#### 2.6.3. Adhesin

**Figure 6:**
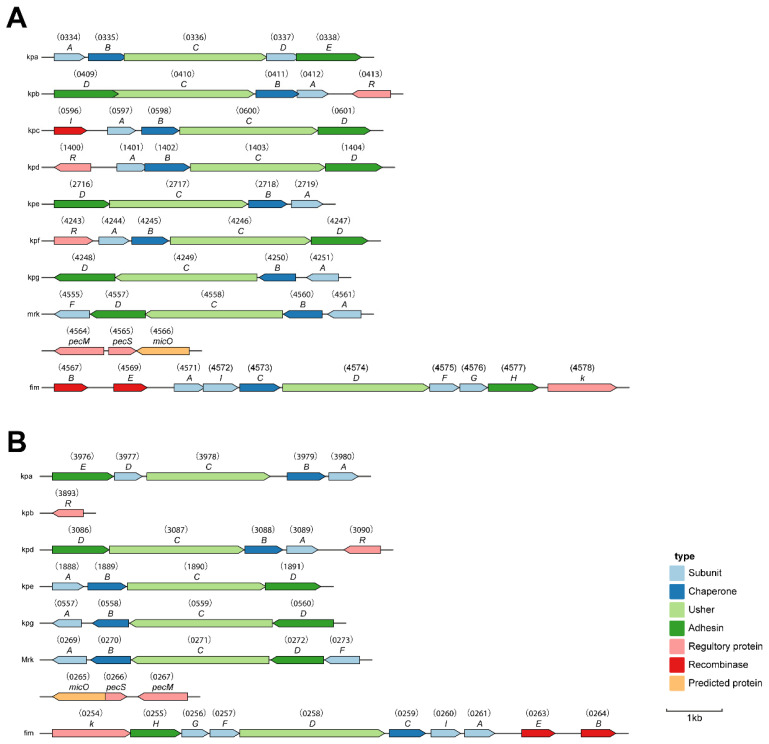
Fimbrial gene clusters of the chaperone–usher-dependent assembly class in *K. pneumoniae* KpC4 and NTUH-K2044. (**A**) NTUH-K2044 [47]. (**B**) KpC4. The locations and orientations of the open reading frames (ORFs) are indicated by arrows. The designation of putative fimbrial genes and the locus tag of ORFs were annotated in the *K. pneumoniae* KpC4 and NTUH-K2044 by KpC4_number and KP1_number, respectively. Each of the putative fimbrial operons is underlined. The putative functions of the ORFs are also shown.

The expression of adhesin plays an important role in the colonization of bacteria and is also the first condition of the pathogenicity of an organism. The adhesive factors of *K. pneumoniae* mainly include type I and Ⅲ fimbriae, and non-fimbrial adhesion protein (Figure 6). Fimbriae are related to bacterial adhesion and colonization of pathogenic processes of *K. pneumoniae*. Currently, nine gene clusters, including *fim, mrk* encoding type I and III fimbriae, and seven (*kpa*, *kpb*, *kpc*, *kpd*, *kpe*, *kpf*, and *kpg*) gene clusters recently discovered, have been identified to be related to the biosynthesis of *K. pneumoniae* fimbriae. Each gene cluster contains at least four genes, which respectively encode a chaperone–usher-dependent assembly system including the fimbrial protein, molecular chaperone, usher protein, and adhesin required for fimbriae biosynthesis. Most of the genes encoding adhesins can be found in the genomes of KpC4 and its close relatives (Additional Table S2).

KpC4 encodes a complete genetic sequence of the type I fimbriae gene cluster (*fimBEAICDFGH*, KpC4_0254-KpC4_0264). Studies have shown that these gene products enhanced *K. pneumoniae* toxicity in urinary tract infections. KpC4 also carries the *fimK* (KpC4_0254) gene not found in *E. coli*, which is located downstream of the *fimH*. Type I fimbria could not express if *fimK* was deficient [44,45]. Type Ⅲ fimbriae encoded by the *mrk* gene cluster (*mrkABCDF*, KpC4_0269-KpC4_0273) can mediate the biofilms formation of *K. pneumoniae* on biotic and abiotic surfaces (such as the catheter in a hospital environment) [46]. This gene structure also includes homologs, including *pecM* (KpC4_0267), *pecS* (KpC4_0266), and *nicO* (KpC4_0265), which are conservative in KpC4, MGH78578, NTUH-k2044, and Kv342 [17,47,48].

**Figure 7:**
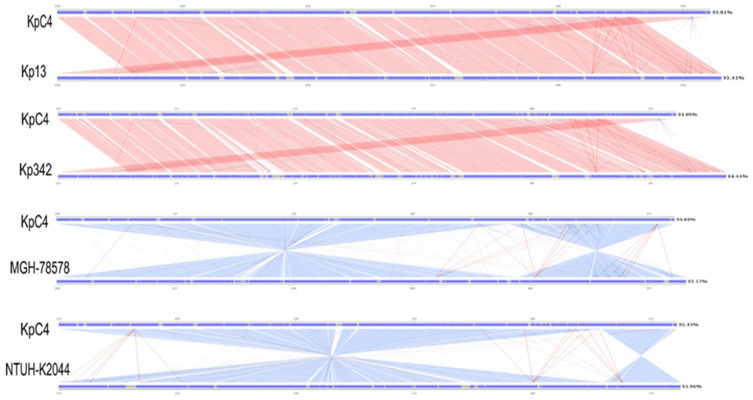
Comparative analysis of genome structures between KpC4 and the reference strains Kp13, Kv342, MGH 78578, and NTUH-K2044.

Additionally, *Kpa*, *kpd*, *kpe*, and *kpg* gene clusters, which are related to the expression of these virulence factors [47] were found in the genomes of both KpC4 and NTUH-K2044 except type I (*fim*) and type III (*mrk*) adhesins. After comparison and analysis, *kpc* and *kpf* gene clusters were not found in the genome of KpC4 (Figure 7), only transcriptional regulatory proteins (KpC4_3893) were encoded by the *kpb* gene clusters, while these six gene clusters were conservative in the genomes of NTUH-K2044, MGH78578, 342, and KP13. Further comparative genomic analysis revealed that NTUH-K2044 and Kp13 possess nine complete fimbriae gene clusters. No gene homologous with *kpc* was found in KpC4, MGH78578, and 342. Wu et al. [47] analyzed 105 *K. pneumoniae* clinical strains with epidemiology and found that the expression of induced recombinants with the *kpc ABCD* genes in *E. coli* resulted in the formation of fimbriae and increased biofilm formation, revealing that the *kpc* gene was highly correlated with the K1 serotype, which is consistent with the experimental results in the literature that KpC4 [12] and MGH78578 (K52) [48] are not of the K1 serotype. Notably, *kpf* gene clusters, found in clinical strains MGH78578, K2044, and Kp13, were absent in both KpC4 and 342. *K. variicola* and *K. pneumoniae* are closely related sister species, and both environmental and clinical strains have extremely similar phenotypic and biochemical characteristics. The 342 (actually *K. variicola* [20,49], nitrogen-fixing endophyte) and KpC4 (crossing-kingdom pathogen infecting plants and animals) strains have unique ecological niches. Therefore, whether the expression of *kpf* is related to different ecological niches or the pathogenicity of clinical strains needs further study.

In addition, studies have shown that CDSs (poly-β-1,6-Nacetyl-D-glucosamine (PGA) adhesion, KpC4_3932-KpC4_3935) encoding the synthesis and transport of PGA adhesin in the KpC4 genome are essential for the stability of the *E. coli* structure [50]. Experiments showed that the regulatory factor of *E. coli* high adhesion phenotype, YidE [51] (KpC4_4342), was related to the survival and proliferation of pathogenic bacteria in the host of the sepsis mouse model [52]. The two-component system *barA/uvrY* contributes to the biofilm formation of *Salmonella enterica*, which is the virulence factor for *E. coli* urinary tract infections and was found in the KpC4 genome (KpC4_0410/KpC4_1149). The *luxS* (KpC4_0525) and luxR family protein (KpC4_1038), encoded the synthesis of autoinducer-2 signaling molecules of *K. pneumoniae* quorum sensing, which were not only important for the biofilm formation, but also conducive to the colonization of pathogens in the host and the improvement of antibiotic resistance [53].

### 2.7. Siderophores and Transporters

Iron is a necessary element for bacterial growth. At least twelve iron uptake systems of *K. pneumoniae* have been confirmed, including four main categories: Fe^2+^ transporters, ABC transporters, iron blood carrier absorption systems, and iron carrier absorption systems. Among them, the ABC transporter Kfu [54], Sit [55], and siderophores systems (Yersinia high-pathogenicity island [56], Iuc and IroA [57]) have proved to be essential for the virulence of *K. pneumoniae* (Table 4).

KpC4 has a complete iron transport system (Additional Table S3). *Feo* (ferrous iron transport) gene clusters (KpC4_4659-KpC4_4660) related to Fe^2+^ capture were found in the KpC4 genome. *SitABCD* system (KpC4_0468-KpC4_0471) related to the transport of bivalent cations such as Mn^2+^ and Fe^2+^, *fecBDE* gene clusters (KpC4_1409-KpC4_1411) related to ferric citrate transport, and *Kfu* gene clusters (KpC4_2567-KpC4_2569) all belong to the ABC transport protein family. It is worth mentioning that the *Kfu* gene cluster encoded by KpC4 is a highly pathogenic island; the *iuc* and *iroA* regions are highly correlated with the HvKP (hypervirulent *K. pneumoniae*) strains; *iroN* (outer membrane siderophore receptor, KpC4_2314) and *IroE* (salmochelin siderophore protein, KpC4_1881) are present in the genome of KpC4. This means that KpC4 has the potential to obtain virulence sites horizontally during the mutation from opportunistic pathogenicity to high virulence [58]. Although the *iucABCD* gene cluster only existed in plasmids of NTUH-K2044 (KP1_p319–KP1_p314), another ferric cell receptor (KpC4_2459) of KpC4 showed 99% similarity to the aerobactin receptor *iutA* (BLASTP [SwissProt:P14542]), which could also replace the function of the *iucABCD* gene cluster and bind exogenous chelating compounds (Additional Table S3).

Enterobactin (Ent) has the highest iron affinity compared to other iron chelators. The *entABCDEF* gene cluster (KpC4_2951-KpC4_2954, KpC4_2964, KpC4_2953) encoded by the genome of KpC4 is essential for Ent biosynthesis, and the *fep* gene cluster (KpC4_4005, KpC4_2963, KpC4_2955, KpC4_2957-KpC4_2959) is responsible for the transport of these siderophores. While the *FhuABCD* system (KpC4_3423-KpC4_3425, KpC4_3427) is involved in the absorption of ferrichrome, the ferrous heme transporter protein HmuRSTUV (KpC4_0458-KpC4_0462) can also be found in the KpC4.

### 2.8. Comparative Genome Analysis

Linear analysis of KpC4, Kv342, and clinical strains MGH 78578, NTUH-K2044, and Kp13 was conducted using SBV (https://github.com/genedenovo/SBV, accessed on 13 June 2020)) software. Comparison of the genomes reveals that the gene structure of KpC4, Kv342, MGH 78578, NTUH-K2044, and Kp13 were highly conserved (Figure 7). However, the region of 4 Mb and 1 Mb (1-3816441, 3772923-5218784) in the KpC4 genome was reversed from the region of the MGH 78578 genome (5315120-3887253, 3896387-1). The same thing happened with NTUH-K2044. KpC4 was highly similar to the clinical strains Kp13 and Kv342, and the collinearity was also good.

The closer the evolutionary sequences, the greater the similarity (including sequence, structure, function, etc.). Except for the similarity of gene composition, the consistency of the sequence of genes on chromosomes in different genomes can better reflect the common origin of genomes. Genetic collinearity would be destroyed by various factors during evolution. The farther the evolutionary distance between species, the worse the genetic collinearity. The degree of collinearity between two species can be used as a measure of the evolutionary distance between them. Apparently, the strains of KpC4 and Kv342, MGH 78578, NTUH-K2044, and Kp13 have high similarity and evolutionary closeness. KpC4 not only has high similarity with Kv342 and Kp13, but also has good collinearity and higher degree of common origin.

### 2.9. Potential Drug Target of K. pneumoniae KpC4

The genomic data of the microbes is conducive to the identification of putative drug targets. These majority drug target candidates (Additional Table S4) which participate in essential processes such as fatty acids, LPS, peptidoglycan, pyrimidine deoxyribonucleotides, and purine nucleotide biosynthesis pathways of KpC4 were identified. It provides more efficient drug targets and therapeutic methods for ever-increasing multidrug-resistant *Klebsiella* and decreasing the available antimicrobial drug activity.

## 3. Discussion

Huang et al. [12] proved with a large number of experiments that *K. pneumoniae* is ubiquitous and able to survive in a variety of harsh environments for a long time, with a strong ecological adaptability. *K. pneumoniae* is not only a pathogen of humans and animals, but it can cross-kingdom infect plants [9]. Ecological adaptability is a survival strategy of *K. pneumoniae* and drives the ecological mechanism by which *K. pneumoniae* infects plants [12].

Here, the whole genome sequencing of KpC4 and the function analysis of some genes provide clues for further exploring the mechanism of cross-kingdom infection of *K. pneumoniae* in plants. KpC4 contains genes associated with survival, colonization, and capability of obtaining living energy materials in the host. Abundant *K. pneumoniae* strains have been isolated from the surface of vegetables [8,59,60,61], and KpC4 also can adhere to the surface of maize plants and seeds [12]. Here, genome sequencing provides evidence that the capsule, type I and Type Ⅲ fimbriae, and adhesin play an important role in KpC4 adhesion to plant surfaces, forming biofilms, and surviving. The biofilm formation is conducive to the adhesion and colonization of *K. pneumoniae* on plant surfaces [62]. Then, KpC4 enters the maize plant through wounds and natural orifices. Genome analyses identified that KpC4 encodes numerous genes involved in the metabolism of carbohydrate and aromatic compounds that can metabolize the cell wall components of plant hosts. The metabolism of these substances can not only promote KpC4 massive colonization and proliferation in plants, provide the material energy required for the basic life activities of KpC4, but also result in plant tissue segregation, decay, and disease. At the same time as it colonizes in the maize plant, KpC4 can protect itself from plant defense mechanisms (ROS, NO, and phytoalexins) by a powerful enzyme system. Among these, thirteen genes (twelve in Kv342, plant endophytic nitrogen-fixing bacterium [17]) encoded glutathione S-transferase (GST) against ROS; two genes (one in Kv342 [17]) encoded flavodoxin reductase, three genes encoded an anaerobic nitrate reduction operon against free radical NO. Additionally, phytoalexin can be excreted extracellular through multidrug transport protein and MDR efflux pump. This genome information implies KpC4 has stronger endophytic living ability than Kv342. This evidence is mutually confirmed with the fact that KpC4 can lead to the natural occurrence of maize top rot in the field, with typical symptoms of leaf margin incision and top rot [9]. 

Most noteworthy, KpC4 encodes plant pathogenic factors which include thirteen genes with highly similarity in nucleotide sequences (identity ≥ 70%) to the pathogenic genes of plant pathogens, and harbors virulence factors that have been shown to be virulent to humans and animals, such as capsular polysaccharide, lipopolysaccharide, adhesin, siderophores, antibiotic resistance and multiantibiotic-related efflux pumps. It has been demonstrated that *K. pneumoniae* strains from different sources have the same pathogenicity on mice [9,63,64,65,66], and the clinical strain Kp138 (K1 serotype) and environmental strain E4 (K1 serotype) also have a similar virulence on maize [9,12]. *K. pneumoniae* appears to use the same strategy to infect different hosts [12]. Our gene functional analysis also supports this view. For example, among the pathogenic genes of KpC4 which caused human/animal disease, the *rcsB* gene encoded a transcriptional regulator of capsular polysaccharide synthesis, which is also the key pathogenic gene of *E. amylovora* and *E. stewartii*, which can cause pear fire blight exhibiting bacterial ooze and maize bacterial wilt, respectively [67,68,69]. *rcsB* plays a vital role in promoting the synthesis of capsular polysaccharide and the biofilm of *E. amylovora and E. stewartia* [69]. Likewise, the main phenotypic characteristic of KpC4 infection in maize, bacterial ooze which mainly composed of capsular polysaccharide, has been observed overflowing from the whorls of the maize [9]. It can be deduced that capsular polysaccharide is the virulence factor, and *rcsB* is the key pathogenic gene for KpC4 cross-kingdom infection. *K. pneumoniae* can use same strategies to infect mammals and plants, which can make itself survive in the organisms of distinct kingdoms after leaving the human/animal host or environment. It not only expands the host range but increases the adaptability. It is the way of inexpensive cost on evolution in which it accelerates the cycle for pathogens from humans to the environment [70].

## 4. Materials and Methods

### 4.1. Strain Isolation and Verification

*Klebsiella pneumoniae* KpC4 was originally isolated as a plant pathogenic bacterium from the interior stems and leaves of maize bacterial top rot disease naturally infected in the fields of many areas of Yunnan Province, China [9]. Strain KpC4 was verified as *K. pneumoniae* using colony morphology and cultural characteristics, Gram-staining test, microscopic examination, physiological and biochemical tests, as well as 16S rRNA, *rpoB*, and *gyrB* amplification in molecular taxonomy [9].

### 4.2. Isolation and Purification of DNA for Library Production

Bacterial cultures were grown on Luria Bertani (LB) medium followed by the isolation of genomic DNA using the Bacterial DNA Kit D3350-01 from OMEGA.

### 4.3. Genome Sequencing

Genome sequencing was carried out using single molecule real-time (SMRT). KpC4 was sequenced and analyzed by Guangzhou Gene Denovo biotechnology Co., LTD., Guangzhou, China. The sequencing process mainly includes sample quality inspection, library building, BluePippin fragment screening, and on-machine sequencing.

### 4.4. Gene Prediction and Annotation

Genome was predicted by GeneMarkes (prokaryotic genome prediction software) to obtain detailed gene distribution and structure information. RepeatMasker software was used to predict the repeat sequence of the genome, rRNAmmer [71] software to predict rRNA, and tRNAscan software to predict the tRNA region and the secondary structure of tRNA. The predicted gene sequences were blast compared with various databases to obtain the protein with the highest similarity to the given gene sequence, and the protein functional annotation information.

### 4.5. Prediction and Functional Analysis of Disease-Related Genes

A BLAST (basic local alignment search tool, ftp://ftp.ncbi.nlm.nih.gov/blast/executables/blast+/LATEST/, accessed on 8 June 2020) local comparing library was built in Windows 10 platform. The protein sequences of KpC4 were used as database files for homology comparison, and 80% coverage, E < 10^−5^, and 90% identities were used as reference standards. When the protein sequences of different genera were searched, the identities of E < 10^−5^, 60% or more was considered that the gene and the matched gene had homology. When E > 10^−5^, the strain was unique and had no homology with other bacteria. Proteins translated from different databases were analyzed, and disease-related genes were manually searched for statistical analysis. Some key genes were compared with closely related *K. pneumoniae* clinical strains (MGH 78578, NTUH-K 2044, Kp13) and *K. variicola* 342 which is an endogenous nitrogen-fixing strain (Kv342). MGH 78578 (accession number: CP000647), a multiple drug-resistant MDR strain, isolated from pulmonary infection patients [52]; NTUH-K2044 (AP006725), hypermucoviscous, was isolated from patients with liver abscesses and meningitis [72]; Kp13 (CP003999) was isolated from the blood of patients with diabetes and cranial injury in the ICU ward [18]. Kv342 (NC011283) is an endophytic nitrogen-fixing bacteria with attenuated pathogenicity to mice [73], which had been misclassified as *K. pneumoniae* because of the relatively similar physiological and biochemical characteristics and genes sequences [74].

## 5. Conclusions

In the study, we determined that *K. pneumoniae* KpC4 had the ability of cross-kingdom infection of plants while maintaining the virulence factors of animal pathogens from the perspective of genomics. Like other *K. pneumoniae* isolates, KpC4 encodes virulence factors such as capsular polysaccharide, lipopolysaccharide, adhesin, siderophores, antibiotic resistance and multiantibiotic-related efflux pumps, which have been shown to be virulent to humans and animals. Intriguingly, KpC4 also harbors plant pathogenic factors which include thirteen genes with high similarity in nucleotide sequences (identity ≥ 70%) to the pathogenic genes of plant pathogens. KpC4 has the molecular basis to survive in animals, environment, and even in plants. Our previous studies also proved that the strong ecological adaptability of KpC4 was the ecological basis of its cross-kingdom infection [12], which opens a whole new area of biological research. The survival mechanism makes KpC4 thriving and prosperous in plants, virulence factors that cause plant disease, same or different strategies to infect organisms in different biospheres, and the mechanisms that cause cross-kingdom infection, are the key hotspots of future research. This information might supplement our understanding of the mechanisms of *K. pneumoniae* cross-kingdom infection. It also laid the foundation for similar microbial evolution studies. The gene function of *rcsB*, and other plant disease-causing genes will be investigated further in our laboratory.

## Figures and Tables

**Table 1:** General features of the KpC4 genome and related analysis.

Kp Strains	Size (bp)	GC Content (%)	No. of CDSs	Mean CDS Size (bp)	Coding Density (%)	No. of tRNAs	Reference
KpC4	5,218,784	58.87	4912	920	86.25	86	This study
MGH 78578	5,315,120	57.5	4776	958	86.1	86	[15]
Kp13	5,307,003	57.5	5288	896	89.3	86	[15]
NTUH-K2044	5,248,520	57.7	5130	939	89.4	86	[16]
Kv342 (*K. variicola*)	5,641,239	57.3	5425	915	88.0	88	[17]
Kp52.145	5,438,894	56.4	5314	-	88.0	85	[17]

**Table 2:** Genes homologous with plant pathogens of KpC4.

Query_id	Gene	Identity (%)	Function	Pathogen Species	Accession	Disease Name	Experimental Host
KpC4_4208	*metJ*	96.19	Repressor of the methionine biosynthesis regulon	*Pectobacterium atrosepticum*	Q6CZA0	Rotting of tubers; blackleg disease of the plant stem	Potato
KpC4_4753	*rsmB*	70.09	Modulates plant cell wall degrading enzymes	*Pectobacterium atrosepticum*	Q6D000	Rotting of tubers; blackleg disease of the plant stem	Potato
KpC4_4136	*corA*	89.87	Magnesium/nickel/cobalt transporter	*Pectobacterium carotovorum*	E1AP33	Soft rot disease	Celery/carrot
KpC4_3550	*RsmA*	79.41	Post-transcriptional regulator	*P. wasabiae*	D0KML5	Soft rot	Tobacco/potato
KpC4_0446	*rpoS*	95.76	Regulation of stress and starvation response	*E. amylovora*	D4HX24	Fire blight	Pear/loquat
KpC4_0922	*rcsB*	92.09	Promote transcription of the genes for capsule synthesis	*E. amylovora*	P96320	Fire blight	Pear
KpC4_4860	*nlpI*	86.05	Tetratricopeptide lipoprotein	*E. amylovora*	D4ICB6	Fire blight	Pear
KpC4_3140	*AcrB*	79.57	Multidrug efflux pump	*E. amylovora*	Q7WTQ9	Fire blight	Apple
KpC4_4819	*rpoN*	84.07	Sigma factor, regulating essential virulence gene	*E. amylovora*	D4HUY5	Fire blight	Apple
KpC4_4766	*AcrB*	79.57	Multidrug efflux pump	*E. amylovora*	Q7WTQ9	Fire blight	Apple
KpC4_4696	*argD*	78.71	N-acetylornithine aminotransferase enzyme	*E. amylovora*	D4I307	Fire blight	Pear/apple
KpC4_0520	*rsmAXoo*	82.14	RNA-binding protein	*Xanthomonas oryzae* pv. *Oryzae*	E2J5T5	Bacterial leaf blight	Rice
KpC4_2459	*iutA*	75.93	Siderophore-mediated iron acquisition	*Pantoea stewartii*	H3RJF2	Stewart wilt of sweet corn	Maize

**Table 3:** Annotation of open reading frames (ORFs) of lipopolysaccharide biosynthesis (lps) loci in KpC4.

Gene Cluster	ORF No.	ORF Name	ORF Location	ORF Homolog Characteristics
***waa***		*coaD*	KpC4_4461	Phosphopantetheine adenylyltransferase
	1	*waaE*	KpC4_4462	Glucosyl transferase
	2	*waaA*	KpC4_4463	Kdo transferase
	3	*ORF4*	KpC4_4464	Glycosyl transferase
	4	*wabH*	KpC4_4465	Glycosyl transferase
	5	*wabG*	KpC4_4466	Glucuronic acid transferase
	6	*waaQ*	KpC4_4467	Heptosyl III transferase
	7	*wabN*	KpC4_4468	Deacetylase
	8	*ORF8*	KpC4_4469	LPS 1,2-N-acetylglucosaminetransferase
	9	*waaL*	KpC4_4470	O-antigen ligase
	10	*ORF10*	KpC4_4471	Glycosyltransferase
	11	*waaC*	KpC4_4472	Heptosyltransferase I
	12	*waaF*	KpC4_4473	Heptosyltransferase II
	13	*hldD*	KpC4_4474	ADP-L-glycero-D-manno-heptose-6-epimerase
		*kbl*	KpC4_4475	2-amino-3-ketobutyrate coenzyme A ligase
***Lpx***		*lpxA*	KpC4_3397	UDP-N-acetylglucosamine acyltransferase
		*lpxB*	KpC4_3396	Lipid-A-disaccharide synthase
		*lpxC*	KpC4_3497	UDP-3-O-acyl N-acetylglucosamine deacetylase
		*lpxD*	KpC4_3399 KpC4_4042	UDP-3-O-(3-hydroxymyristoyl)-glucosamine n-acyltransferase
		*lpxK*	KpC4_2629	Tetraacyldisaccharide 4′-kinase

**Table 4:** Iron uptake systems of KpC4 and related *Klebsiella*.

Category	System	Gene	Role	CDS ^∆^	Find In				
					KpC4	78578	K2044	Kp13	342
Feo	Feo	*feoABC*	Fe^2+^ transport	KpC4_4660	+	−	+	−	−
ABC transporter	Sit	*sitABCD*	Fe^2+^ transport	KpC4_0468	+	+	+	+	+
	Kfu	*kfuABC*	Fe^3+^ transport	KpC4_2569					
	Fec	*fecBDE*	Ferric citrate transport	KpC4_1411					
		*fpbABC*	Fe^3+^ transport		−	−	+	−	+
Hemophore-based	Hmu	*hmuRSTUV*	Heme utilization	KpC4_0458	+	+	+	+	+
Siderophore-based	Fep	*fepABCG*	Enterobactin transport	KpC4_4005	+	+	+	+	+
	Ent	*EntA-F*	Enterobactin synthesis	KpC4_2951	+	+	+	+	+
	Fhu	*fhuA-C*	Ferrichrome transport	KpC4_3427					
	IroA	*iroN*	Salmochelin transport	KpC4_2314	−	−	+	−	−
		*iroBCDE*	Salmochelin synthesis	KpC4_1881	‡	−	+	−	−
	Aerobactin	*iutA*	Aerobactin transport	KpC4_2459	+	±	+	±	±
		*iucABCD*	Aerobactin synthesis		−	−	+	−	−
	Yersinia HPI	*ybtPQXS, ybtA-irp2-irp1- ybtUTE-fyuA*	Yersiniabactin synthesis and transport		−	−	+	+	−

∆, initial CDS of the system relative to the KpC4 genome; −/+, absent/present in this strain; ‡, IroE appears truncated in this strain; ±, a CDS sharing 70% identity is found, although the rest of the system was not identified.

## Data Availability

All the data is available in the manuscript and in Supplementary section. The sequencing information is deposited into the GenBank under BioProject number: PRJNA338238.

## Supplementary Materials

The following supporting information can be downloaded at: https://www.mdpi.com/article/10.3390/ijms232416005/s1.
