# Supplementary material for: Novel Virulence Factors Deciphering Klebsiella pneumoniae KpC4 Infect Maize as a Crossing-Kingdom Pathogen: An Emerging Environmental Threat
Source: Int J Mol Sci. 2022 Dec 15;23(24):16005. doi: 10.3390/ijms232416005 (PMC9785288; doi:10.3390/ijms232416005)
Supplement: Supplementary file 1 [file ijms-23-16005-s001.zip › ijms-2029427-supplementary.pdf]

International Journal of *Molecular Sciences*

MDPI

Supplementary Data and Additional Files

Novel Virulence Factors Deciphering *Klebsiella pneumoniae* KpC4 Infect Maize as a Crossing-Kingdom Pathogen: An Emerging Environmental Threat

Additional Table S1. Annotation of open reading frames (ORFs) of capsular polysaccharide synthesis (*cps*) loci in KpC4.

ORF no.	ORF name	ORF location	ORF homolog characteristics
1	*galF*	KpC4_1048	UTP–glucose-1-phosphate uridylyltransferase
2	*ORF2*	KpC4_1049	acid phosphatase
3	*wzi*	KpC4_1050	capsule assembly protein
4	*wza*	KpC4_1051	polysaccharide export protein
5	*wzb*	KpC4_1052	protein-tyrosine-phosphatase
6	*wzc*	KpC4_1053	tyrosine protein kinase
7	*wbaP*	KpC4_1054	Undecaprenyl-phosphate galactose phosphotransferase
8	*ORF7*	KpC4_1055	glycosyl transferase
9	*ORF8*	KpC4_1056	glycosyl transferase/amylovoran biosynthesis protein AmsE
10	*ORF9*	KpC4_1057	glycosyl transferase
11	*ORF10*	KpC4_1058	glycosyl transferase
12	*ORF11*	KpC4_1059	O-antigen and lipid-linked capsular repeat unit polymerase
13	*ORF12*	KpC4_1060	IS630 family transposase
14	*ORF13*	KpC4_1061	glycoside hydrolase
15	*ORF15*	KpC4_1062	O-antigen flippase
16	*gnd*	KpC4_1063	Gluconate-6-phosphate dehydrogenase
17	*ugd*	KpC4_1064	UDP-glucose 6-dehydrogenase 尿嘧啶雙磷酸葡萄糖去氫酶
I	*wzy*	KpC4_4152	common antigen polymerase
II	*wecF*	KpC4_4153	4-alpha-L-fucosyltransferase
III	*wzx*	KpC4_4154	O-antigen translocase

Additional Data from Table S2. Adhesins gene cluster of KpC4 and related *Klebsiella.*

Gene cluster	Description	CDS$^{\Delta}$	Found in				
			KpC4	K2044	78578	342	Kp13
matB	Fimbrillin matB homolog	KpC4_3300	+	+	+	+	+
fim	fimbrial protein fimK	KpC4_0254	+	+	+	+	+
	fimbrial protein fimH	KpC4_0255	+	+	+	+	+
	type 1 fimbrial protein fimG	KpC4_0256	+	+	+	+	+
	type 1 fimbrial protein fimF	KpC4_0257	+	+	+	+	+
	Outer membrane usher protein fimD	KpC4_0258	+	+	+	+	+
	Molecular chaperone FimC	KpC4_0259	+	+	+	+	+
	Fimbrin-like protein fimI	KpC4_0260	+	+	+	+	+
	type-1 fimbrial protein subunit A	KpC4_0261	+	+	+	+	+
	Type 1 fimbriae regulatory protein FimE	KpC4_0263	+	+	+	+	+
	Type 1 fimbriae regulatory protein fimB	KpC4_0264	+	+	+	+	+
mrk	Type III fimbrial protein mrkA precursor	KpC4_0269	+	+	+	+	+
	Type III fimbriae chaperone MrkB	KpC4_0270	+	+	+	+	+
	Fimbrial protein MrkC	KpC4_0271	+	+	+	+	+
	Fimbrial protein mrkD	KpC4_0272	+	+	+	+	+
	type III fimbriae protein mrkF	KpC4_0273	+	+	+	+	+

Additional data from Table S2. Continued.

Gene cluster	Description	CDS$^{\Delta}$	Found in				
			KpC4	K2044	78578	342	Kp13
kpa	Fimbrial protein	KpC4_3980	+	+	+	+	+
	Fimbrial assembly protein	KpC4_3979	+	+	+	+	+
	Fimbrial biogenesis outer membrane usher protein	KpC4_3978	+	+	+	+	+
	Fimbrial morphology protein	KpC4_3977	+	+	+	+	+
	Fimbrial protein	KpC4_3976	+	+	+	+	+
kpb	Putative transcriptional regulator protein	KpC4_3893		+	+	+	+
	Probable adhesin		−	+	+	+	+
	Outer membrane usher protein		−	+	+	+	+
	FimC-like chaperone protein		−	+	+	+	+
	Fimbrial protein		−	+	+	+	+
kpc	Recombinase involved in phase variation		−	+	−	−	+
	Fimbrial protein		−	+	−	−	+
	Putative fimbrial chaperone protein precursor		−	+	−	−	+
	Long polar fimbrial outer membrane usher		−	+	−	−	+
	putative fimbrial adhesin precursor		−	+	−	−	+
kpd	Fimbrial protein	KpC4_3086	+	+	+	+	+
	Outer membrane usher protein	KpC4_3087	+	+	+	+	+

Additional Data from Table S2. Continued.

Gene cluster	Description	CDS$^{\Delta}$	Found in				
			KpC4	K2044	78578	342	Kp13
	Fimbrial chaperone	KpC4_3088	+	+	+	+	+
	Fimbrial protein	KpC4_3089	+	+	+	+	+
	Putative transcriptional regulator	KpC4_3090					
kpe	Putative fimbrial-like protein	KpC4_1888	+	+	+	+	+
	Fimbrial protein/Chaperone protein	KpC4_1889	+	+	+	+	+
	Fimbrial protein	KpC4_1890	+	+	+	+	+
	Fimbrial protein	KpC4_1891	+	+	+	+	+
kpf	Fimbrial subunit type 1 precursor		−	+	+	**± (58%)**	+
	pilus assembly protein		−	+	+	**± (60%)**	+
	Outer membrane usher protein		−	+	+	−	+
	Putive fimbrial protein		−	+	+	−	+
kpg	Fimbrial protein	KpC4_0560	+	+	+	+	+

Gene cluster	Description	CDS$^{\Delta}$	Found in				
			KpC4	K2044	78578	342	Kp13
	Export and assembly usher protein of type 1fimbriae	KpC4_0559	+	+	+	+	+
	fimbrial assembly protein	KpC4_0558	+	+	+	+	−
	Fimbrial protein	KpC4_0557	+	+	+	± (69%)	+

Δ, locus of the CDS in KpC4. +, gene found with BLASTP identity ≥ 90% and at least 80% coverage. −, gene absent according to the same criteria. ±, presence of a similar sequence, but which did not pass the constraints (% BLASTP similarity).

Additional Data from Table S3. Iron scavenging systems of KpC4 and related *Klebsiella.*

Category	System	Gene	Gene products	Role	CDS^Δ	Find in				
						KpC4	78578	K2044	Kp13	342
**Feo**	Feo	*feoABC*	Fe(2+) transporter permease subunit FeoB	Fe^{2+} transport	KpC4_4660	+	–	+	–	–
			ferrous iron transporter C		KpC4_4659	+	–	+	–	–
**ABC transporter**	Sit	*sitABCD*	metal ABC transporter permease	Fe^{2+} transport	KpC4_0468	+	+	+	+	+
			metal ABC transporter permease		KpC4_0469	+	+	+	+	+
			manganese/iron transporter ATP-binding protein		KpC4_0470	+	+	+	+	+
			metal ABC transporter substrate-binding protein		KpC4_0471	+	+	+	+	+
	Kfu	*kfuA*	iron(III)-binding periplasmic protein	Fe^{3+} transport	KpC4_2569	+	+	+	+	+
		kfuB	*iron(III)-transport system permease*		KpC4_2568	+	+	+	+	+
		kfuC	*iron(III)-transport ATP-binding protein*		KpC4_2567	+	+	+	+	+
	Fec	*fecB*	iron(III) dicitrate transport ATP-binding protein	Ferric citrate transport	KpC4_1411	+	+	+	+	+
		fecD	iron(III) dicitrate transport system permease		KpC4_1410	+	+	+	+	+
		fecE	iron(III) dicitrate transport ATP-binding protein		KpC4_1409	+	+	+	+	+
		fpbABC		Fe^{3+} transport		–	–	+	–	+
**Hemophore based**	Hmu	*hmuRSTUV*	heme ABC transporter ATP-binding protein	Heme utilization	KpC4_0458	+	+	+	+	+

Additional Data from Table S3. Continued.

Category	System	Gene	Gene products	Role	CDS^Δ	Find in				
						KpC4	78578	K2044	Kp13	342
**Hemophore based**	Hmu	*hmuRSTUV*	iron ABC transporter permease		KpC4_0459	+	+	+	+	+
			hemin ABC transporter substrate-binding protein		KpC4_0460	+	+	+	+	+
			hemin-degrading factor		KpC4_0461	+	+	+	+	+
			TonB-dependent receptor		KpC4_0462	+	+	+	+	+
**Siderophore based**	Fep	*fepA*	outer membrane receptor FepA		KpC4_4005<br>KpC4_2963	+	+	+	+	+
		fepB	ferric enterobactin (enterochelin) binding protein		KpC4_2955	+	+	+	+	+
		fepC	Ferric enterobactin transport ATP-binding protein		KpC4_2959	+	+	+	+	+
		fepD	ferric enterobactin transport system permease protein		KpC4_2957	+	+	+	+	+
		fepG	ferric enterobactin transport system permease protein		KpC4_2958	+	+	+	+	+

Category	System	Gene	Gene products	Role	CDS^Δ	Find in				
						KpC4	78578	K2044	Kp13	342
	Ent	*entA*	2,3-dihydro-2,3-dihydroxybenzoate dehydrogenase		KpC4_2951	+	+	+	+	+

**Siderophore based**		*entB*	2,3-dihydro-2,3-dihydroxybenzoate synthetase	Enterobactin synthesis	KpC4_2952	+	+	+	+	+
		entC	isochorismate synthase		KpC4_2954	+	+	+	+	+
		entD	enterochelin synthetase component D		KpC4_2964	+	+	+	+	+
		entE	2,3-dihydroxybenzoate-AMP ligase		KpC4_2953	+	+	+	+	+
		entF	ATP-dependent serine activating enzyme		KpC4_2960	+	+	+	+	+
	Fhu	*fhuA*	ferrichrome porin	Ferrichrome transport	KpC4_3427	+	+	+	+	+
		fhuB	iron(3+)-hydroxamate import system permease		KpC4_3423	+	+	+	+	+
		fhuC	ferrichrome ABC transporter, ATP-binding protein		KpC4_3425	+	+	+	+	+
		fhuD	iron(3+)-hydroxamate-binding protein		KpC4_3424	+	+	+	+	+
	IroA	*iroN*	Outer Membrane Siderophore Receptor	Salmochelin transport	KpC4_2314	+	–	+	–	–

Additional Data from Table S3. Continued.

Category	System	Gene	Gene products	Role	CDS$^{\Delta}$	Find in				
						**KpC4**	**78578**	**K2044**	**Kp13**	**342**
**Siderophore based**	IroA	*iroE*	Salmochelin siderophore protein IroE	Salmochelin synthesis	KpC4_1881	+	–	+	–	–
	Aerobactin	*iutA*	Outer membrane receptor proteins, mostly Fe transport	Aerobactin transport	KpC4_2459	+	±	+	±	±
		iucABCD		Aerobactin synthesis		–	–	+	–	–
	High-patho-genicity island	*ybtPQXS, ybtA-irp2-irp1- ybtUTE-fyuA*		Yersiniabactin synthesis and transport		–	–	+	+	–

Δ, CDS of the iron scavenging systems system relative to the KpC4 genome; +, gene found with BLASTP identity ≥ 90% and at least 80% coverage. −, gene absent according to the same criteria; ±, a CDS sharing 70% identity is found, although the rest of the system was not identified.

Additional Data from Table S4. The potential targets of *K. pneumoniae* KpC4 Continued.

No.	CDS	Gene	Product	Essentiality	Functional pathway	Proposed inhibitors
1	KpC4-0844	*FabB*	3-oxoacyl-[acyl-carrier-protein] synthase 1	Yes	Type II fatty acid elongation cycle	TLM, cerulenin
2	KpC4_0324	*ArgA*	Amino acid acetyltransferase	Yes	Catalyze the first step in L- arginine biosynthesis	
3	KpC4_3397	*LpxA*	UDP-N-acetylglucosamine O- acyltransferase	Yes	Catalyze the first-step of lipid IV(A) LPS biosynthesis	Compounds 1 and 2
4	KpC4_3497	*LpxC*	UDP-3-O-[3- hydroxymyristoyl] N- acetylglucosamine deacetylase	Yes	Catalyze the committed step in lipid A LPS biosynthesis	LpxC-2, LpxC-3, LpxC-4
5	KpC4_2478	*FabH*	3-oxoacyl-[acyl-carrier-protein] synthase 3	No	Catalyze the first condensation reaction in fatty acid biosynthesis	Small molecule inhibitors
6	KpC4_3412	*DapD*	2,3,4,5-tetrahydropyridine- 2,6-dicarboxylate N- succinyltransferase	Yes	Catalyze the first step of the L-lysine biosynthesis via DAP pathway	L-2-aminopimelate
7	KpC4_3508	*MurF*	UDP-N-acetylmuramoyl-tripeptide–D-alanyl-D-alanine ligase	Yes	Peptidoglycan biosynthesis	Thiophene-3-carbonitriles
8	KpC4_0724	*DapE*	Succinyl- diaminopimelate desuccinylase	Yes	Produce intermediates required for L-lysine biosynthesis	L- Captopril
9	KpC4_3399<br>KpC4_4042	*LpxD*	UDP-3-O-[3-hydroxymyristoyl] glucosamine N-acyltransferase	Yes	Cell membrane glycolipid biosynthesis	RJPXD33
10	KpC4_2270	*FabI*	Enoyl-[acyl-carrier-protein] reductase [NADH]	Yes	Enoyl reductase involved in biotin and fatty acids biosynthesis	Diazaborines, triclosan
11	KpC4_3396	*LpxB*	Lipid- A- disaccharide synthase	Yes	Biosynthesis of lipid A disaccharide	antisense pPNA

Additional Data from Table S4. The potential targets of *K. pneumoniae* KpC4 Continued.

No.	CDS	Gene	Product	Essentiality	Functional pathway	Proposed inhibitors
12	KpC4_3504	*MurG*	Undecaprenyl-PP-MurNAc- pentapeptide-UDPGlcNAcGlcNAc transferase	Yes	Peptidoglycan biosynthesis	Substituted prolines
13	KpC4_1800	*Asd*	Aspartate- semialdehyde dehydrogenase	Yes	Biosynthesis of multiple components; L-lysine, L-threonine, L-methionine, L- homoserine	2-Chloro-1,4-naphthoquinone, 2-Bromo-1,4-naphthoquinone
14	KpC4_2425	*PurB*	Adenylosuccinate lyase	Yes	Biosynthesis of purine nucleotides	-
15	KpC4_2471	*Tmk*	Thymidylate kinase	Yes	Biosynthesis of pyrimidine deoxyribonucleotides	-
16	KpC4_3504	*MurG*	UDP-N-acetylglucosamine–N- acetylmuramyl-(pentapeptide) pyrophosphoryl- undecaprenol N-acetylglucosamine transferase	Yes	Cell wall formation and Lipid synthesis	Substituted prolines
17	KpC4_1153	*SecA*	Protein translocase subunit SecA	Yes	Protein translocation across the bacterial membrane	Azide, decatransin
18	KpC4_3508	*MurF*	UDP-N-acetylmuramoyl- tripeptide-d-alanyl-d-alanine ligase	Yes	Cell wall formation and murein synthesis	Thiophene-3-carbonitriles, Cyanothiophene derivatives

19	KpC4_4467	*WaaQ*	Lipopolysaccharide core biosynthesis protein	Yes	LPS biosynthesis	-

Additional Data from Table S4. The potential targets of *K. pneumoniae* KpC4 Continued.

No.	CDS	Gene	Product	Essentiality	Functional pathway	Proposed inhibitors
20	KpC4_1093	*SbcB*	Exodeoxyribonuclease I	Yes	Degrades single-stranded DNA	-
21	KpC4_0080	*TolC*	Outer membrane channel protein	Yes	Bacterial efflux system, export antibiotics and other toxic compounds	Domperidone
22	KpC4_3229	*YajC*	Preprotein translocase subunit	Yes	Protein secretion, insertion of proteins into membranes, and assembly of membrane protein complexes	-
